# Species determination using AI machine-learning algorithms: *Hebeloma* as a case study

**DOI:** 10.1186/s43008-022-00099-x

**Published:** 2022-06-30

**Authors:** Peter Bartlett, Ursula Eberhardt, Nicole Schütz, Henry J. Beker

**Affiliations:** 1 La Baraka, Gorse Hill Road, Virginia Water, Surrey, GU25 4AP UK; 2 Staatliches Museum für Naturkunde Stuttgart, Rosenstein 1, 70191 Stuttgart, Germany; 3 Rue Père de Deken 19, 1040, Bruxelles, Belgium; 4 Royal Holloway College, University of London, Egham, UK; 5 Plantentuin Meise, Nieuwelaan 38, B-1860 Meise, Belgium

**Keywords:** Agaricales, Ectomycorrhizal fungi, Identification keys, Taxonomy, Neural networks

## Abstract

The genus *Hebeloma* is renowned as difficult when it comes to species determination. Historically, many dichotomous keys have been published and used with varying success rate. Over the last 20 years the authors have built a database of *Hebeloma* collections containing not only metadata but also parametrized morphological descriptions, where for about a third of the cases micromorphological characters have been analysed and are included, as well as DNA sequences for almost every collection. The database now has about 9000 collections including nearly every type collection worldwide and represents over 120 different taxa. Almost every collection has been analysed and identified to species using a combination of the available molecular and morphological data in addition to locality and habitat information. Based on these data an Artificial Intelligence (AI) machine-learning species identifier has been developed that takes as input locality data and a small number of the morphological parameters. Using a random test set of more than 600 collections from the database, not utilized within the set of collections used to train the identifier, the species identifier was able to identify 77% correctly with its highest probabilistic match, 96% within its three most likely determinations and over 99% of collections within its five most likely determinations.

**Supplementary Information:**

The online version contains supplementary material available at 10.1186/s43008-022-00099-x.

## INTRODUCTION

### Delineating species within *Hebeloma*

Why define species and what makes a species are questions that continue to be a matter of debate among biologists. Many of the discussed species concepts are in practice difficult to test (Taylor et al. 2000; De Queiroz 2007; Hey 2001, 2006; Hey et al. 2003), or are theoretical concepts rather than operational definitions. In the quest for species limits, usually a discontinuation of characters is sought, be it reproductive isolation, physiological, ecological, geographical traits, morphological or genetic discordance or a combination of several of these. In practice, the currently most popular criterium (Matute and Sepúlveda 2019; Lücking et al. 2020) for the delimitation of species is reciprocal monophyly, ideally observed in several independent genetic markers analysed independently (genealogic concordance). Also, not so rarely, reciprocal monophyly only in a single genetic marker, currently usually the ITS^1^ (Schoch et al. 2012; Lücking et al. 2020), is used for delimitation. Dettman et al. (2003) pointed out that in cases in which reproductive isolation precedes genetic divergence, biological species may not be recognizable by (reciprocal) monophyly. Although species are still being described morphologically, in practice species identification is often done primarily based on DNA sequence data, even when morphology is available, owing to its speed and the reproducibility of the result. However, whenever possible morphology is still used as a touchstone when testing the validity of molecularly (or otherwise) defined taxa, both in terms of the similarity within the defined group as well as for distinguishing its members from the members of other taxa.

Species delimitation is often not easy in *Hebeloma*. Following He et al. (2019) and Miyauchi et al. (2020), it appears to be one of the younger genera of the Agaricales. Many species share the same general appearance, size range and colouring. Also, molecularly, some species tend to be rather variable intraspecifically and not very divergent interspecifically (e.g. Aanen and Kuyper 1999; Eberhardt et al. 2015; Grilli et al. 2016).

*Hebeloma*, certainly in Europe, is one of the Agaricales genera the taxonomy and nomenclature of which have been investigated in depth in the past decade. Recent descriptions made from molecularly tested collections exist for all European and a number of non-European species. Within our work (see Beker et al. 2016), we have used various techniques to investigate the taxonomy of *Hebeloma* species and their boundaries. For the molecular delimitation of species, we have explored various regions of the genome and for morphological species delimitation, we have made use of a sophisticated database which currently has the parametrized details of about 9000 *Hebeloma* collections. Reproductive isolation cannot be tested in *Hebeloma.* Many species have never been grown in culture, and even where crosses of monokaryotic strains succeeded, basidiomes, i.e. basidiospores, were not formed by the resulting dikaryotic mycelia. For the assignment to putative biological species we have relied heavily on the work of Aanen et al. (2000a, 2000b, 2001, 2004). We have followed the ideas of De Queiroz (2007) in considering ‘lines of evidence’ with regard to species delimitation and searching for evidence using a combination of the three hypotheses mentioned above as well as searching for any ecological and biogeographical evidence.

### Database

Our starting point for the delineation of species is the *Hebeloma* Database. This database, hosted on Biolomics version 12 from Bioaware SA NV, contains information of about 9,000 collections of *Hebeloma* from around the world. The set of collections includes all holotypes, epitypes, neotypes and lectotypes, as well as many isotypes and paratypes, that could be located and loaned for study. The database entries contain collection details and a morphological description, as a number of parameters describing the collection both macroscopically and microscopically; for about a third of the cases, micromorphological characters have been analysed and are included. This allows searches of the database collections based on a given set of parameters. In this way, collections with similar properties have been clustered, parameters of collections that fall into the same phylogenetic clades have been compared and single-access keys (for example those published in Beker et al. (2016)) have been built on the database in the form of queries that are continually tested against all the database collections.

The Biolomics database does not, however, form a critical part of the identifier tool developed here. The user may supply data about collections to the tool in the simpler form of a comma-separated values (CSV) file.

### Morphological characters

The characters used within the study have been refined from a list that various authors (for example Bruchet 1970; Romagnesi 1965, 1983; Favre 1955, 1960; Boekhout 1982; Smith et al. 1983; Vesterholt 1995, 2005) had developed over a period of years. Throughout the process of developing a tool for species identification, there is a need to balance a number of competing factors: choosing characters that actually have the power to distinguish between species, choosing characters for which sufficient data is available to train an algorithm and choosing characters that will be ‘user-friendly’ when others come to use the identifier in the future.

For those collections collected by the authors, the methodology for morphological analysis followed was that described in Beker et al. (2016). Collections we have received from herbaria or that were sent in by other mycologists come with varying amounts of information. Often some parameters had to be estimated from photographs or the exsiccate, which imposes dangers of inaccuracy. While spores are normally relatively well preserved even in old material, cystidia and basidia may be badly collapsed. For type material, macroscopic information was taken from the protologue, while microscopic analysis was carried out on the material and supplemented from the protologue where necessary. Many characters now known to be important in species separation may not have been recorded by the collector, e.g. many mycologists in the past did not record the number of full-length lamellae, that is, lamellae stretching from the stipe to the pileus edge. Similarly, the awareness of the mycorrhizal relationship between *Hebeloma* and various trees and shrubs varied over time. While collectors of the more distant past may not have noted possible host plants at all, others may only have noted the closest possible associate. For older collections, photographs rarely exist.

For each analysed collection, subject to the state of the material, about 50 spores were measured in Melzer’s reagent, excluding the apiculus. The maximum length and width of each spore was measured and its Q value (ratio of length to width) calculated. Average length, width and Q value were calculated and recorded alongside the median, standard deviation and 5% and 95% percentiles. The assessment and coding of spore characters followed Beker et al. (2016) and Vesterholt (2005).

The average width of the widest part of the cheilocystidium in the vicinity of the apex appears to be an important character in the separation of species within *Hebeloma* (Vesterholt 2005). As written before, it is also important, when determining this average width near the apex, not to be selective with regard to the cystidia chosen for measurement. To determine the average width at the apex about 100 cheilocystidia (where possible) were measured on the lamellae edge. For other measurements, at least 20 cheilocystidia (again, where possible), separated from the lamella edge, were measured from each collection. Because of the complex shapes of the cheilocystidia, four measurements were made: length, width at apex (A), width at narrowest point in central region (M) and maximum width in lower half (B). For each cheilocystidium, the ratios A/M, A/B and B/M were also calculated and the average values of the four measurements and three ratios across all measured cheilocystidia were recorded. Measurements were made in 5% KOH and Melzer’s reagent. For all other details with regard to our methodology, see Beker et al. (2016).

Among the species accepted by Beker et al. (2016) only two pairs of species (*H. aanenii* and *H geminatum*; *H. album*, syn. *H. fragilipes* and *H. pseudofragilipes*) that could not be unambiguously identified morphologically were accepted.

### Sequence data

The generation of ITS sequence data was attempted for almost all of the collections examined, dependent on permission. The internal transcribed spacer (ITS) region of the nuclear ribosomal RNA genes has been established as ‘the’ species marker for fungi (Kõljalg et al. 2005; Schoch et al. 2012). This marker system has been shown to err more often on the ‘lumping’ than on the ‘splitting’ side, i.e. the species resolution of the ITS is not always sufficient (Schoch et al. 2012; see also the “ITS caveats” of Lücking et al. 2020). This is certainly true for *Hebeloma*. Additional genetic markers were then amplified and sequenced from a subset of collections, aiding the molecular phylogeny and delimitation of species and helping with species identification.

Within our project we have embraced in various combinations two variable domains of the mitochondrial small subunit of the ribosomal RNA genes (mitSSU), referred to as V6 and V9, as well as part of the gene for the second largest subunit of RNA polymerase II (*RPB2*), a partial sequence of the translation elongation factor 1 α (*TEF1a*) and the protein coding gene *MCM7* (Minichromosome Maintenance Complex Component 7). Technical details can be found in Eberhardt (2012), Eberhardt et al. (2016, 2021a), Vesterholt et al. (2014) and Cripps et al. (2019) and references therein.

In most publications from the project, Maximum Likelihood analyses of single and concatenated genetic markers were used to explore the molecular support of species, ideally by reciprocal monophyly of species clades in several genetic markers analysed separately, thus meeting the prerequisites of Taylor et al. (2000) “Genealogical Concordance Phylogenetic Species Recognition (GCPSR)”. In practice, a number of species could not be recognized this way. In Beker et al. (2016), the term “non-discordant” was used for cases, when all sequences of a species were paraphyletic in relation to (a) monophyletic sister taxon, but not mixed with sequences from other species.

At the time Beker et al. (2016) was published, 64 of the 84 species accepted had at least five sequences for the majority of genetic markers used (ITS, *TEF1a*, *RPB2*, V6 and V9 of the mitSSU). Twenty-four of these 64 species were monophyletic in all five genetic markers. Eight species were recognized based on morphology, ecology and (if available) putative reproductive isolation, but did not form monophyla in any of the single marker results; five of these taxa not even in the phylogram of the concatenated dataset (one of these species, *H. oreophilum*, we no longer recognize as distinct from *H. clavulipes*, also in Beker et al. 2016, see below).

### Establishment of species boundaries

Armed with these data, agreement was sought between the molecular and morphological analysis in order to help fix species boundaries, also using locality and habitat data as well as biological mating group data, where that existed. With these boundaries fixed, a total of 123 distinct species have to-date been described using this methodology: 79 of the 84 species from Beker et al. (2016) are included as-is. The remaining five have been synonymised in later work–*H. fragilipes* is replaced by *H. album*, *H. dunense* is replaced by *H. velatum,* both *H. oreophilum* and *H. clavulipes* are replaced by *H. palustre* (all Eberhardt et al. 2022a) and *H. hygrophilum* is replaced by *H. paludicola* (Eberhardt et al. in prep). Thirty-one further species have been published as new or confirmed recently from Europe and other continents (Monedero and Alvarado 2020; Grilli et al. 2020; Eberhardt et al. 2020a, 2020b, Eberhardt et al., 2021a, 2021b, 2022a, 2022b). The remaining nine species, from Mexico and the United States, will be discussed in two forthcoming papers (Eberhardt et al. in press, in prep.). The complete list is given in Table 1. Note that this list does not cover all *Hebeloma* names currently accepted, only those that are current and have been described using the methodology discussed above.

**Table 1:** Current names of analysed and accepted *Hebeloma* species, by section

*Hebeloma*	Includes
sect. *Adherentia*	*H. adherens*
‘section Australe’	*H. australe*
sect. *Denudata,* subsect. *Clepsydroida*	*H. album, H. ammophilum, H. asperosporum, H. cavipes, H. cinnamomeum, H. ingratum, H. laetitiae, H. limbatum, H. matritense, H. pseudofragilipes, H. sordidulum, H. vaccinum*
sect. *Denudata,* subsect. *Crustuliniformia*	*H. aanenii, H. alpinum, H. arcticum, H. aurantioumbrinum, H. bellotianum, H. crustuliniforme, H. eburneum * *H. geminatum, H. helodes, H. louiseae, H. luteicystidiatum, H. lutense, H. magnicystidiatum, H. minus, H. pallidolabiatum, H. perexiguum, H. pusillum, H. salicicola*
sect. *Denudata,* subsect. *Echinospora*	*H. echinosporum, H. populinum, H. rostratum*
sect. *Denudata*, subsect. *Hiemalia*	*H. hiemale*
sect. *Duracinus*	*H. duracinoides*
sect. *Hebeloma,* ‘subsect1’	*H. fuscatum, H. grandisporum, H. monticola, H. nigellum, H. paludicola, H. palustre, H. sordescens, H. spetsbergense*
sect. *Hebeloma,* ‘subsect2’	*H. alpinicola, H. ambustiterranum, H. cistophilum, H. colvinii, H. excedens, H. harperi, H. marginatulum, H. mesophaeum, H. pascuense, H. psammophilum, H. pubescens, H. subtortum, H. velatum*
‘section Islandica’	*H. islandicum*
‘section Mediorufa’	*H. lacteocoffeatum, H. mediorufum, H. nothofagetorum*
sect. *Myxocybe*	*H. radicosum, H. sagarae*
sect. *Naviculospora*	*H. avellaneum*, *H. catalaunicum, H. nanum, H. naviculosporum, H. subaustrale, H. subfastibile*
sect. *Porphyrospora*	*H. aminophilum, H. angustilamellatum, H. flavidifolium, H. ifeleletorum, H. indicum, H. lactariolens, H. parvisporum, H. porphyrosporum, H. radicans, H. sarcophyllum, H. victoriense, H. vinosophyllum, H. youngii*
sect. *Pseudoamarescens*	*H. pseudoamarescens*
sect. *Sacchariolentia*	*H. fusisporum, H. ischnostylum, H. nauseosum, H. odoratissimum, H. sacchariolens*
sect. *Scabrispora*	*H. anthracophilum*, *H. birrus*, *H. circinans*, *H. cylindrosporum*, *H. danicum*, *H. laterinum*, *H. lindae*, *H. luchuense*, *H. melleum*, *H. pumilum*, *H. radicosoides*, *H. viscidissimum*
sect. *Sinapizantia*	*H. bulbiferum*, *H. sinapizans*
sect. *Syrjense*	*H. syrjense*
sect. *Theobromina*	*H. alboerumpens, H. cohaerens*, *H. erumpens*, *H. griseopruinatum*, *H. parvicystidiatum*, *H. plesiocistum, H. theobrominum, H. vesterholtii*
sect. *Velutipes*	*H. aestivale, H. albidulum, H. celatum, H. citrisporum, H. erebium, H. incarnatulum, H. leucosarx, H. neurophyllum, H. quercetorum, H. subconcolor, H. velutipes*

### Identification keys

In the monograph of Beker et al. (2016), 84 European species of *Hebeloma*, within 13 sections and four subsections, were described and dichotomous identification keys provided to sections, subsections and species within sections. The descriptions and the keys were built on the basis of the database collections for each species. Consequently, for species for which the database contained many collections from different locations and habitats it should be expected that the intraspecific variation was well covered, while for those species with very few collections, possibly rarer species, the descriptions given would necessarily be limited.

The identification keys provided in Beker et al. (2016) were polytomous keys, allowing the user two or more options at each choice, although in most cases only two options were allowed (dichotomous). Such keys are usually referred to as single-access keys (e.g. Hagedorn et al. 2010) or in computer jargon, decision trees. With such keys the author defines a fixed set of choices (decisions) which the user must follow. At every step the user must choose between two (or occasionally more) choices and decide which path to take. At any stage a wrong decision will lead to either a wrong answer or to a dead-end where none of the options presented apply. For non-interactive keys, such as those printed in a book, single-access keys are the most common.

The keys of Beker et al. (2016) were tested as a set of database queries across all collections of the 84 recognized European species. At the time of writing the monograph, the database had about 4500 records, that is, an average of about 50 collections of each species. However, in practice, while some species were represented by more than 200 collections others had very few collections, in a small number of cases only one. Some of the southern European species were at the time under-represented on the database; this has since been largely rectified in Grilli et al. (2020). This book highlighted a number of instances where, with a larger database of collections, it was clear that some of the species descriptions in Beker et al. (2016) had been too narrow and consequently the dichotomous keys provided were inadequate and could lead to incorrect determinations. While pointing out these difficulties associated with descriptions based on a small number of collections, Grilli et al. (2020) did not provide updated keys.

Multi-access keys (often called ‘synoptic’ keys, although some authors reserve the term synoptic for keys that strictly follow a taxonomic path rather than focusing on diagnostic features that aid species separation) allow the user, at every step, to select from a list of characters and choose which to address, rather than having to follow a predefined and fixed path through the characters. Multi-access keys lend themselves to computer-aided use.

Multi-access keys, particularly those that are interactive, have a number of advantages over single-access keys. The user, trying to determine the species of their specimen, may select and enter information in any order; focusing on features (characters) and questions for which they believe they have the information to correctly answer the question. The computer can present additional information or guidance as each character is addressed. After every step, the user may be presented with possible identifications and even further guidance including images or supplemental text.

### Generalising identification keys with machine learning

In recent years the concepts of Artificial Intelligence (AI) and machine-learning (ML)^2^ have gained traction. AI encompasses the concept of developing computer systems able to perform tasks normally requiring human intelligence, such as visual perception, speech recognition, decision-making and translation between languages. ML algorithms are used in a wide variety of applications, such as in medicine, fraud recognition, image recognition and email filtering, where it is difficult to develop explicit algorithms to perform the tasks. In the case of identification keys, the idea is to generalize the concept of a multi-access key. Instead of a human expert devising one key, a computer is allowed to search among a very large number of possible keys and select one that accurately assigns the most sample data, known as “training data”, to the correct species. Thus, the predictions or decisions are made without being explicitly programmed to do so. Moreover, the algorithm will also include uncertainty or probabilistic estimates to reduce the prospect of being sent down a “blind alley” as is possible particularly with single access keys. It is also anticipated that as the training data expands so the computer model automatically adapts to the new information received, rather than requiring a human to revisit a key authored by hand.

The use of AI and ML in species determination is still in its infancy but evolving rapidly as advances in the fundamental ML algorithms are developed by computer scientists. Within mycology, we are aware of two broad approaches to the use of ML tools: image-based learning and sequence-based learning.

For images, an example is using macroscopic images. Zieliński et al. (2020) used a deep learning approach to classify microscopic images of various fungus species found in the context of human infection. Evangelisti et al. (2021) used deep learning to quantify the extent and kind of root colonization and hyphae abundance of arbuscular mycorrhizal fungi via images of plant root systems.

For sequences, Vu et al. (2020) applied a deep learning approach to the classification of yeast and mould barcode sequences. Delgado-Serrano et al. (2016) developed a Naïve Bayes classifier to classify to genus level based on ITS1 sequences. Meher et al. (2019) created a fungal classifier again using ITS sequences, but used a random forest approach to machine learning.

Outside of mycology, a broader overview of machine learning approaches to species determination is given in Wäldchen and Mäder (2018), however the focus is exclusively on image-based machine learning. The image-based approach is commonly applied to plants (see e.g. Sun et al. 2017; Mahmudul Hassan and Kumar Maji 2021; Bambil et al. 2020).

**Fig. 1:**
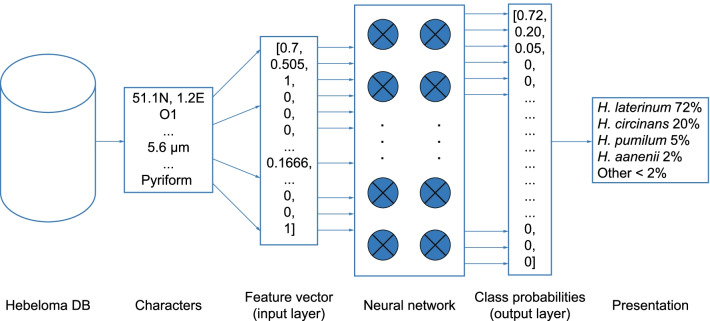
Conceptual workflow for a character-based machine learning identifier. Characters are transformed to feature vectors which are transformed into probabilities by the machine. Finally, these are mapped back to classes (species or sections) for presentation

The uniqueness in this present study is the application of ML algorithms not to images or sequence data but to macro- and microscopic character data of a single fungal genus (Fig. 1). The machine “learns” the characters that together are characteristic of a particular species. We present here the manner in which both a ‘training set’ of sample data and a ‘testing set’ of collections were selected. The testing set is not used at all during the training phase and is used only to assess the accuracy of the algorithm once it has been calibrated. The parameters that were used in training are discussed, together with results from different experiments that were carried out using different parameters.

Additionally, not every collection has all character data defined–many collections came from herbaria, citizen scientists or mycologists with incomplete data. While, in most cases, the microscopic information could be generated, much of the macroscopic information or metadata is lost forever. It is useful however to include even incomplete collection data as it may aid the effectiveness of the algorithm–a method to include such data is presented. As well as availability, the subjectivity (and thus consistency) in the measurement of the character is also important. For instance, colour, given so much variation in conditions, age and perception, may be particularly inconsistently recorded. A discussion of which groups of characters were useful is included.

## MATERIALS AND METHODS

A *Hebeloma* species was recognised for this study if it had been publicly described and analysed using the methods outlined in the introduction. The full list of 123 recognised species is shown in Table 1, ordered by (sub)section. The groupings used largely follow the thirteen sections and four subsections of Beker et al. (2016) but with the following additions:

- *H.* sect. *Adherentia*, introduced in Monedero and Alvarado (2020).
- The placement of *H. islandicum*, originally included within *H.* sect. *Naviculospora* in Beker et al. (2016), was found to be problematic, as discussed in Eberhardt et al. (2021a). Here it is treated as being in a separate section, to which we refer as ‘section Islandica’.
- *H. australe*, not found in Europe and thus not treated in Beker et al. (2016), was found to ‘sit’ outside the thirteen defined sections. Consequently, it is here treated as being within a separate entity, to which we here refer as ‘section Australe’.
- Similarly, the New Zealand species of *H. lacteocoffeatum, H. mediorufum, H. nothofagetorum* are referred to here as ‘section Mediorufa’.
- *H.* sect. *Hebeloma* is divided into two informal ‘subsections’ based on morphology: ‘subsect1’ and ‘subsect2’. Section *Hebeloma* ‘subsect1’ consists of those species with distinctly dextrinoid, mainly amygdaloid spores and ‘subsect2’ consists of those species with indextrinoid or at most indistinctly dextrinoid, mainly ellipsoid to ovoid spores.

It is our intention to properly define the new sections and subsections in future publications.

A collection is included in the study if it had been assigned to one of these included species and if it has been analysed such that sufficient of its characters are available. Of the 8928 collections on the database, 3072 met both of these conditions and so were used. The remainder were not used in the study. Within the calibration of each identifier, 70% of collections for each species were assigned, at random, to the training set and 30% to the testing set. These sets will be defined further below.

For each tested group of characters, two identifiers were created–one that is trained on species and thus assigns (probabilistically) a collection to a species, and one that is trained only on the (sub)section to which a training set collection belongs and thus assigns to a (sub)section too. We call these a “species identifier” and “(sub)section identifier” respectively. Although any species identifier can be turned into a (sub)section identifier by simply summing probabilities of species in the same (sub)section it was decided to test the direct training on sections as the two approaches would not necessarily give the same result.

### Machine learning from sets of characters

Viewed from the perspective of machine learning (ML), the problem of assigning a collection parameterized by a set of characters to a species, can be viewed as a multi-class supervised learning problem. In the terminology of ML, the characters are the “features” and the species or (sub-)sections are the “classes”. By “supervised” it is meant that the machine is told to which class particular collections, those in the training set, belong.

Suppose that, given a collection *c* and a set of characters F, the collection can be represented by a vector of numbers F(c). This vector is called the feature vector. The mechanism for determining this representation is discussed later. Further suppose that the possible class of outputs is S (in this case study S will always be the set of *Hebeloma* species or the set of *Hebeloma* sections and subsections) which has M elements. Then a machine learning algorithm is a function *NN* that takes a vector of length N as the input and outputs a vector length M, where each element of the output is between 0 and 1 and sum up to 1. The *i-th* element of the output is interpreted as the probability that collection *c* under consideration belongs to the *i-th* class:

$${\text{P}}\left( {\text{c,i}} \right) = {\text{ NN}}\left( {{\text{F}}\left( {\text{c}} \right),learnable \, parameters} \right)\left[ {\text{i}} \right]$$

Suppose the *actual* class of the collection is C. The *cross-entropy loss* of this function is then defined to be:

$$\mathop \sum \limits_{{{\text{i}} \in S}} - {\text{I}}_{{\left\{ {{\text{i}} = {\text{C}}} \right\}}} \log \left( {{\text{p}}\left( {{\text{c}},{\text{i}}} \right)} \right)$$

where *I* is the indicator function. This loss is high if the probability assigned to the correct species is low and monotonically tends towards zero as the probability tends to 1. Very low probabilities are particularly highly penalized due to the nature of the log function. Let the total loss be the sum of the cross-entropy losses across all collections in the training set. The calibration of a machine learning algorithm is equivalent to finding the parameters in NN that minimize the total loss across all members of the training set: if the total loss is small, then all training set collections must have a high probability of being assigned to the correct species.

Note it is assumed there is no uncertainty to the expert determination that placed collection *c* in class C. Nor is there any uncertainty in the measurement of characters (i.e. in the value of F(c)). This implies, from the perspective of the machine learning algorithm, that these collections and their assignments are seen as the “gospel” truth that we are trying to learn from.

The choice of NN for the name of the function belies the fact this function is usually chosen to be a particular type of function called a neural network. Such functions can be minimized by iteratively updating the learnable parameters by a process of back propagation (Chauvin and Rumelhart 1995). Once the loss function is minimized, the learnable parameters are frozen and the function NN is completely known.

The feature vectors of other collections, i.e. collections not from the training set, can then be computed and the function NN applied to these new vectors. A set of class probabilities is then obtained for the new collection. If the machine is well-chosen (i.e. if the training set and testing set have similar properties and the character group is rich enough to detect these similarities), then these probabilities will also match the actual class of the new collection. If, on the other hand, the function NN has good results for the training set but not other collections, then the function is said to be ‘overfitted’.

### Choosing a ML framework

While the learnable parameters are varied as the iterative solver searches for a solution, there are several ways to control the functional form of the function NN and how the solver iterates. Collectively these can be seen as hyperparameters to the system. In this study we experiment with various hyper-parameterizations and report on the results.

The base software used was Python 3.7.8 (Van Rossum and Drake 2009) primarily developed and run on a Windows 10 laptop with typical hardware specifications, but also tested on Linux. The scripts we developed were dependent on the following open source Python packages: PyTorch (Paszke et al. 2019) version 1.9.1, scipy v1.5.2 (https://scipy.org/), pandas v0.25.1 (https://pandas.pydata.org/), numpy v1.19.2 (https://numpy.org/), dill v0.3.2 (https://pypi.org/project/dill/) and reverse_geocoder v1.5.1 (https://pypi.org/project/reverse_geocoder/), but did not depend on these specific versions in a critical way; other versions may be substituted. Interaction with the tool for both creating and verifying identifiers is carried out via a command line interface.

Each functional form NN is implemented as a PyTorch *torch.nn.Module* instance with either one or two hidden layers. Each hidden layer has an activation function of either Rectified Linear Unit (ReLU) (Agarap 2018) or Mish (Misra 2020). The dimensionality of each hidden layer was set equal to the maximum of the dimensionality of the feature set and the dimensionality of the class.

A total of five optimizers were used to minimize the loss function: Stochastic Gradient Descent (SGD) (Bottou 1999), Adam (Kingma and Ba 2015) and AdamW (Loshchilov and Hutter 2019). For both the Adam and AdamW optimizers, the “AMSGrad” variation proposed in Reddi et al. (2018) was also evaluated.

For Adam and AdamW, an identifier was calibrated for each learning rate in the set {0.0001, 0.0002, 0.0005, 0.001, 0.002, 0.005, 0.01, 0.02, 0.05} and the highest performing identifier chosen. For SGD learning rates {0.05, 0.1, 0.2} were used. In every case the optimizer search was allowed to run for a maximum of 1000 epochs.

For all identifiers that were created, the pool of available collections was always divided so that, within each class, 70% of the collections were assigned to the training set and 30% to the testing set. Within a class, the collections were assigned to the training or testing set at random using Python’s built-in *random* module. In particular there was no attempt to balance the two sets so that each contained collections with any particular property. Critically, as with all machine learning, *only* the training test was used to calibrate the learnable parameters of the identifier. The testing set was set aside and used only to test the calibrated identifier on an independent set of data.

### Encoding characters to features

In the preceding discussion it was supposed for each collection that there was a vector F(c) of length N, where N is the dimensionality of the feature set to use as input to the machine learning algorithm. In practice however the data is not initially in numeric form. E.g. the notions “Was associating with *Pinaceae*” or “Has spore ornamentation type O2” are not numeric. Therefore, prior to passing a collection to the machine learning algorithm, a transformation was first applied. In this case study, characters were always transformed to a value between 0 and 1. Thus a collection of N characters becomes equivalent to a point on or in the N-dimensional [0, 1] cube. The mapping of continuously-valued characters and discretely-valued characters are handled separately.

### Continuously-valued characters

Given a character, there is more than one way to map it to a feature value. For example, average spore lengths might range from 5 µm to 20 µm. A simple way to map is by uniform mapping–5 µm maps to 0, 20 µm maps to 1 with linear interpolation in between. In practice characters are not distributed uniformly–it is much more likely for the average spore length of a *Hebeloma* collection to be around 11 µm long rather than 20 µm. A uniform mapping would therefore cause too much ‘bunching’ around typical feature values. Instead, a probability distribution is implied from collections in the training set and assigned a feature value F(y) = P(X < = y) in the implied distribution. By construction this means that feature values in the training set are ‘spread out’ between 0 and 1. Although ML algorithms should, in principle, be able to handle this re-scaling themselves with a well-chosen network, in practice, it appears that this extra ‘help’ improves the quality of the learning. The implied distributions for all characters are shown in Additional file 1: Appendix Fig. S1.

### Discretely-valued characters

The discretely-valued characters in this study are of the form “Is O3-type spore ornamentation present?” or “Was the collection found in Europe?” and therefore have only two possible values. Thus, the character simply maps to 0 if it is false or absent and to 1 if it is present or true.

### Character groups

The scheme described above shows how each character is mapped to a feature value. Each character is mapped individually and does not depend on any other character that the collection may have. This means that it is possible to take arbitrary groups of characters and create an identifier from that group. In this study nine such ‘character groups’ were chosen manually by experimentation and guesswork as to which groups of characters might work well together. They were also guided by the amount of available data: it was not possible to include a character if very few collections had data recorded for that character. There was no attempt, in this study, to ask the machine to determine automatically a ‘good’ group of characters to use. Table 2 shows which characters were included in which group.

**Table 2:** Characters chosen for each of the tested characters groups

Character	Type	Dims	Character group										
			1	2	3	4	5	6	7	8	9	A	B
Latitude	Continuous geographic	1	✓	✓	✓	✓	✓	✓	✓	✓	✓		
Longitude	Continuous geographic	1	✓	✓	✓	✓	✓	✓	✓	✓	✓		
Altitude	Continuous geographic	1	✓	✓	✓	✓	✓	✓	✓	✓	✓		
Is Europe, Is North America	Discrete geographic	2	Only as filter										
Associates with plant family	Discrete habitat–associating with *Pinaceae, Salicaceae or Fagaceae*	3									✓		
Number of complete lamellae	Continuous macroscopic	1			✓	✓	✓	✓	✓	✓	✓	✓	✓
Spore ornamentation O1–O4	Discrete microscopic	4	✓	✓	✓	✓	✓	✓	✓	✓	✓		
Perispore loosening P0–P3	Discrete microscopic	4	✓	✓	✓	✓	✓	✓	✓	✓	✓		
Spore dextrinoidity D0–D4	Discrete microscopic	5	✓	✓	✓	✓	✓	✓	✓	✓	✓	✓	✓
Spore length	Continuous microscopic	1	✓	✓	✓	✓	✓	✓	✓	✓	✓		
Spore width	Continuous microscopic	1	✓	✓	✓	✓			✓	✓	✓	✓	✓
Spore Q	Continuous microscopic	1				✓	✓		✓	✓	✓		
Cheilocystidia length	Continuous microscopic	1	✓	✓	✓	✓	✓	✓	✓	✓	✓		
Cheilocystidia apex width	Continuous microscopic	1					✓	✓	✓	✓	✓		
Cheilocystidia A/M	Continuous microscopic	1						✓	✓	✓	✓		
Cheilocystidia A/B	Continuous microscopic	1					✓	✓	✓	✓	✓		
Cheilocystidia B/M	Continuous microscopic	1					✓	✓	✓	✓	✓		
Cheilocystidia main shape	Discrete microscopic–Shape is *cylindrical*, *ventricose-lageniform*, *clavate gently*, *clavate-stipitate*, *clavate-ventricose* or *pyriform*	6		✓		✓	✓	✓	✓	✓	✓		
Basidia Q (length/width ratio)	Continuous microscopic	1								✓	✓		✓

Table 2 also shows the number of dimensions each character contributed to the feature vector. For example, collections were characterized as having one of six primary cheilocystidia shapes. Each shape contributed one dimension (“has shape cylindrical”, “has shape pyriform” and so on) for a total of 6 dimensions. The total number of dimensions in the feature vector ranges from as few as 19 for CG1 through to 33 for CG9. Character Groups A and B have far fewer characters included in them. These special character groups will be used only for “second-pass” section-specific identifiers (see “Section-specific identifiers” section below).

### Identifier enhancement techniques

The preceding section discussed the basic workflow for creating an identifier from characters. In this section, extensions to the basic workflow that might be expected to enhance the results that the identifier can achieve are examined.

#### Data augmentation

As discussed, just over 3,000 collections have had a sufficient number of their characters analysed to be of potential use to the algorithm. Even within these collections however, the complete set of data may not have been recorded, e.g. for historical collections, the number of complete lamellae may not be available. The underlying machine learning algorithms always require complete data. That is, a collection can only be included in a testing or training set for a particular Character Group if that collection has data for all characters required by that Group. We are thus left with a dilemma–if we increase the number of characters used by a particular Group, with the aim of giving the machine more information, the available pool of collections with complete data *decreases*, thus reducing the amount of ‘training’ provided.

Because many collections were missing just one character we are able to reduce the impact of the problem by introducing a form of Data Augmentation (Shorten and Khoshgoftaar 2019) that enables the collection to be included in the training set. Specifically, if a collection is missing one character, it was augmented with the average value of that character across other collections of that species in the training set.

#### Section-specific identifiers

Another approach to the problem of limited data is that of chaining different identifiers utilizing the fact that some characters are only important within a subset of species in the genus, typically a subsection. We could use an identifier *I,* using a limited set of characters, to establish to which section a collection is likely to belong and, if that section has collections with more data, apply another identifier, trained only on collections in that section but using more characters, to identify the species within the section. This approach means that complete data for collections is only required for particular sections, rather than for the whole genus.

Even if more data is not available, it seems possible that a dedicated or focused identifier, trained only on the subset, may also give an improved estimate, particularly if the section appears “difficult”. A further possible variation is to use fewer characters in a particular section in order to give greater importance to those that remain. In this study we experiment with *Hebeloma* subsect. *‘*subsect1’ by producing specialised identifiers trained only on the section but keep the character group the same as the ‘parent’ identifier. In particular we will test CGA and CGB, groups consisting of only a small number of characters that our experience suggest are particularly good at distinguishing members of *Hebeloma* subsect. *‘*subsect1’.

The notion of ‘chained’ identifiers is formalized as follows. Given a set of species S and a collection c, suppose that identifier I assigns a probability P_I,s_ (c) that collection c belongs to species s in S. Further suppose there exists a subset of species T ⊂ S and another identifier J that assigns c to t ∈ T with probability P_J,t_(c). Then a new identifier can be defined as I-then-J:

$${\text{P}}_{{{\text{I}} - {\text{then}} - {\text{J}},{\text{s}}}} \left( c \right) = \left\{ {\begin{array}{*{20}c} {P_{I,s} \left( c \right)} & { if\;s \notin T} \\ {{{P_{J,s} \left( c \right)} \mathord{\left/ {\vphantom {{P_{J,s} \left( c \right)} {\mathop \sum \limits_{t \in T} P_{I,t} \left( c \right)}}} \right. \kern-\nulldelimiterspace} {\mathop \sum \limits_{t \in T} P_{I,t} \left( c \right)}}} & { if\;s \in T} \\ \end{array} } \right.$$

i.e. for species within the subset T, the probability from the “specialist” identifier J is assigned, weighted so that the total probability across all species is 1.

#### Post identifier filtering

An identifier will usually assign some probability (however small) to every possible class. We may decide that the collection cannot possibly be from that class due to some particular value that it has. A typical example may be a collection from Europe cannot possibly be a species that is known only in North America. We may choose to assign probability zero to all North America-only species and scale up probabilities for other species appropriately. In this case we call the continent character a filtering character. The decision on whether a character value implies exclusion could be taken completely independently (i.e. by external expert judgement) or could be implied from the training set. In the latter case we would say that a species is “known to North America” if there is a collection of that species in the training set.

The notion of a filtering character is thus potentially a double-edged sword. It may improve the quality of an identifier by filtering out false positive identifications, but also runs the risk of assigning *zero* probability to new discoveries (e.g. a species found on a continent for the first time). This issue is discussed further in the results.

In formal terms, suppose there exists an identifier I which assigns probability P_I,s_(c) to collection *c* being of class s and a feature F which filters out class T ⊂ S if F(c)ϵR– “the exclusion zone”, then the filtered identifier I’ is defined by

$$P_{{I^{\prime},s}} \left( c \right) = \left\{ {\begin{array}{*{20}c} 0 & {if\;s \in T} \\ {{{P_{I,s} \left( c \right)} \mathord{\left/ {\vphantom {{P_{I,s} \left( c \right)} {\mathop \sum \limits_{t \in S\backslash T} P_{I,t} \left( c \right) }}} \right. \kern-\nulldelimiterspace} {\mathop \sum \limits_{t \in S\backslash T} P_{I,t} \left( c \right) }}} & { if\;s \notin T} \\ \end{array} } \right.$$

### Metrics

We evaluated the performance of a calibrated identifier by calculating several commonly-used (e.g. Sammut and Webb 2010) metrics for collections in the testing set.

A collection is said to be of rank K if the identifier assigned the collection’s true class as its Kth highest probability. The metrics are then defined as follows:

*Top N score* The proportion of testing set collections that were rank N or higher. In particular, the “Top 1” score is the proportion of collections where the identifier assigned the highest probability to the correct species. The Top 3 score is the proportion to which the identifier assigned one of its highest 3 probabilities and so on. We give scores for Top 1, Top 3 and Top 5.

*Mean Reciprocal Rank (MRR)* If a collection is Rank N, then assign the score 1/N to this collection. The MRR score is then the average of these reciprocal ranks across the testing set. The highest possible score is 1 (if all collections are Rank 1) so becomes comparable with *Top N* but does not suffer from the problem of an arbitrary cut-off of that metric.

*Macro-averaged F1* $\left( {F_{1}^{m} } \right)$ As described in Picek et al. (2021a), the macro-averaged F1 score (Chinchor 1992) is a metric that removes the bias in favour of classes with large numbers of collections. The authors note that this imbalance of dataset sizes is prevalent in nature and that it is true also in the case of the *Hebeloma* database. We have some species (e.g. *mesophaeum*) with hundreds of collections but other species (e.g. *grandisporum*) with collections in single figures. The overall score $F_{1}^{m} = \frac{1}{N}\sum F_{1,S}$, where N is the number of classes and F_1,S_ is the class-specific score defined by

$$F_{1,S} = 2{{\left( {P_{S} * R_{S} } \right)} \mathord{\left/ {\vphantom {{\left( {P_{S} * R_{S} } \right)} {\left( {P_{S} + R_{S} } \right)}}} \right. \kern-\nulldelimiterspace} {\left( {P_{S} + R_{S} } \right)}}$$, where

$${\text{P}}_{{\text{S}}} = \frac{{TP_{S} }}{{TP_{S} + FP_{S} }}$$

$${\text{R}}_{{\text{S}}} = \frac{{TP_{S} }}{{TP_{S} + FN_{S} }}$$

Here TP_S_ is the number of collections correctly defined as S (true positives, or Top 1 predictions for this class), FP_S_ is the number of collections incorrectly predicted to be S with the highest probability (false positives) and FN_S_ is the number of collections of type S that were predicted to be something else (false negatives). I.e. P_S_ is the precision; R_S_ is the sensitivity; and F_1_ is the harmonic mean of the two.

## RESULTS

**Fig. 2:**
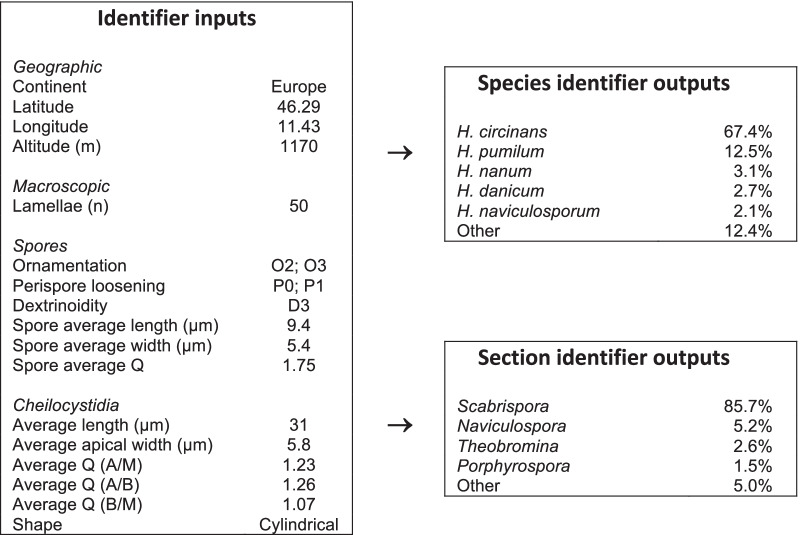
Representation of the identifier as presented to a user. The user enters inputs on the left-hand side. The identifier computes class probabilities by calculating the same Neutral Network function as during the training phase, but now with fixed ‘learnt’ parameters. The data used here is from collection HJB17396. Expert analysis had assessed this collection to be *Hebeloma circinans* from *H.* sect. *Scabrispora*. Thus, in this case, the top ‘guess’ of the identifier is correct and this result would be included in the “Top 1” metric

For each combination of Character Groups and hyperparameters tried, the training phase completed successfully and returned a set of learned parameters. Taken together, each set of Character Group, hyperparameters and learned parameters collectively becomes an identifier that is saved to a file and kept for future use. A user of the identifier supplies as inputs the values of characters in the Character Group and the identifier outputs predicted classes for both species and section, listed in order of probability. A typical set of inputs and outputs for Character Group 7 is shown in Fig. 2.

For each identifier, each metric was computed for the testing set. The most accurate identifier for species classification uses Character Group 8, a neural network with one hidden layer using a ReLU activation function and the AMSGrad variant of the AdamW optimizer. This identifier has a Top 1 metric of 76.7%, a top 3 metric of 95.8% and a Top 5 metric of 99.2%. The MRR score is 86.5 and F_1_ score is 72.1. The corresponding identifier tackling the problem of assigning to the correct section has Top 1, 3 and 5 metrics of 94.9%, 99.3% and 99.9% respectively.

Table 3 shows the results for all character groups with other parameters held fixed. The weakest identifiers–CG2, CG3 and CG1– are the characters groups that omit the number of lamellae and cheilocystidia shape features and both respectively, suggesting these characters are particularly important in allowing the identifier to resolve species. The pattern of adding in further characters to improve the identifier by any metric continues up until CG8. However, the addition of including the plant association character (i.e. moving from CG8 to CG9) causes a decrease in performance. It is important to note that the available training set size was smaller for this character group.

**Table 3:** Species identification success metrics for various choices of characters used to calibrate the classifier

	Testing set	Metrics (/100)				
Character group	size (n)	Top 1	Top 3	Top 5	MRR	F1m
CG1	790	57.3	84.4	92.9	72.1	48.6
CG2	779	66.5	91.8	95.5	79.2	63.2
CG3	678	63.6	91.4	96.2	77.7	57.7
CG4	685	73.3	94.9	97.7	84.2	72.3
CG5	671	74.1	94.9	99.3	84.8	68.5
CG6	671	75.4	94.8	98.5	85.4	70.2
CG7	671	75.0	95.4	98.8	85.2	72.4
CG8	528	76.7	95.8	99.2	86.5	72.1
CG9	469	73.6	94.5	98.5	84.3	69.1

In these results, neither the continent filter nor second pass is applied, and collections without recorded data for at most one character are permitted. The optimization method is AdamW and the network shape is one hidden layer with a ReLU activation function. In this and subsequent results tables, the final five columns refer to the Top 1, Top 3, Top 5, Mean Reciprocal Rank and macro F1 metrics respectively, all scaled to give a score out of 100, which would represent perfect prediction

Additional file 2: Appendix Table S1 shows a complete breakdown, for each species and section, for the CG7 identifier with the hyperparameters as in Table 3. The table reveals that some sections are more difficult to classify than others, with MRR scores ranging from 95 for *Scabrispora* down to 78 for *Denudata-Clepsydroida* amongst sections which had more than 50 collections in their testing set.

Table 4 shows the results for identifiers where the target classes were (sub)sections rather than species. In this case there are only 21 output classes rather than 123 in the case of species. As with the case of species, the CG1, CG2 and CG3 character groups are significantly worse than the others, but the CG4-9 perform broadly similarly.

**Table 4:** Section identification for the same set of characters groups. The hyperparameters were the same as in Table 3

	Testing set	Metrics (/100)				
Character group	size (n)	Top 1	Top 3	Top 5	MRR	F1m
CG1	790	75.2	96.5	99.1	85.6	56.3
CG2	779	88.4	98.5	99.7	93.5	72.4
CG3	678	79.9	97.8	99.4	88.8	67.0
CG4	685	89.8	99.1	99.9	94.5	74.9
CG5	671	94.5	99.6	99.9	97.0	81.0
CG6	671	94.6	99.0	99.9	96.9	80.0
CG7	671	94.9	99.3	99.9	97.2	82.8
CG8	528	94.5	99.4	100.0	97.0	80.0
CG9	469	94.7	99.6	99.8	97.1	82.2

### Extensions

Table 5 compares identifier results in the case where data augmentation was allowed to interpolate missing data in the training set and when it was not. Results are shown for Character Groups 7 and 8; all other parameters were held constant as in Table 3.

**Table 5:** Comparison of results using augmented and non-augmented data

			Metrics (/100)				
Class	CG	Augmentation	Top 1	Top 3	Top 5	MRR	F1m
Species	7	Not augmented	77.1	95.6	99.0	86.5	73.3
Species	7	Augmented	75.0	95.4	98.8	85.2	72.4
Species	8	Not augmented	71.2	93.8	97.6	82.2	64.8
Species	8	Augmented	76.7	95.8	99.2	86.5	72.1
Section	7	Not augmented	94.2	99.7	99.9	96.8	80.0
Section	7	Augmented	94.9	99.3	99.9	97.2	82.8
Section	8	Not augmented	94.9	99.3	99.8	97.1	82.4
Section	8	Augmented	94.5	99.4	100.0	97.0	80.0

The results indicate that adding extra, albeit incomplete, data was helpful for Character Group 8, but not Character Group 7.

Table 6 shows the results of applying a “second pass” identifier on collections that were suspected to be (with probability > = 90%) of being in section *Hebeloma* subsect. ‘subsect1’ by the main or “first pass” identifier. Where the first pass character group 7, most choices for the second pass did not improve results. An exception is using Character Group 8 for the second pass. In this case an improvement was seen for the Top 1, Top 5 and MRR metrics. When the first pass used CG8, no second pass gave an improvement across the majority of metrics.

**Table 6:** Comparison of results where the identifier works in two phases

			Metrics (/100)				
Class	CG	Second pass CG	Top 1	Top 3	Top 5	MRR	F1m
Species	7	No second pass	75.0	95.4	98.8	85.2	72.4
Species	7	1	74.5	94.9	98.5	84.9	71.4
Species	7	2	74.1	94.3	98.7	84.6	71.0
Species	7	3	75.3	95.4	98.8	85.4	72.2
Species	7	4	75.0	95.2	98.7	85.2	72.0
Species	7	5	74.7	95.1	98.7	85.0	71.6
Species	7	6	74.5	95.4	98.8	84.9	71.6
Species	7	7	74.7	95.2	98.7	85.0	72.0
Species	7	8	77.5	95.3	99.2	86.7	72.1
Species	7	9	72.3	94.7	98.3	83.7	66.3
Species	7	A	73.8	94.6	99.0	84.3	70.3
Species	7	B	75.6	93.9	99.1	85.2	69.7
Species	8	No second pass	76.7	95.8	99.2	86.5	72.1
Species	8	1	75.9	95.1	99.2	85.8	71.0
Species	8	2	75.8	95.3	99.2	85.8	70.8
Species	8	3	75.9	95.3	99.2	86.0	71.0
Species	8	4	76.1	95.8	99.2	86.1	70.9
Species	8	5	75.9	96.0	99.4	86.0	71.1
Species	8	6	75.9	95.5	99.1	86.0	71.2
Species	8	7	76.1	96.2	99.4	86.2	70.8
Species	8	8	75.8	95.5	99.2	85.9	70.9
Species	8	9	74.2	95.3	98.9	85.0	69.5
Species	8	A	73.5	93.9	99.1	84.2	69.2
Species	8	B	73.5	94.3	98.7	84.3	69.2

The first phase used Character groups CG7 or CG8 and was applied to the whole genus. The second pass used another Character Group and was only applied to the collections that the first pass suggested had a > = 90% chance of being in *Hebeloma* subsect. ‘subsect1’. The second pass identifiers were trained only on data from *Hebeloma* subsect. ‘subsect1’ collections

Table 7 compares identifier results in the case where post-identifier filtering was applied to exclude species which had not been found on the continent on which the test set collection was found. Character Groups 7 and 8 were used and all other parameters held constant.

**Table 7:** Comparison of results where “continent filtering” was applied

			Metrics (/100)				
Class	CG	Continent filtering	Top 1	Top 3	Top 5	MRR	F1m
Species	7	Not filtered	75.0	95.4	98.8	85.2	72.4
Species	7	Filtered	75.6	95.7	98.8	85.8	73.3
Species	8	Not filtered	76.7	95.8	99.2	86.5	72.1
Species	8	Filtered	77.7	96.2	99.4	87.1	73.5
Section	7	Not filtered	94.2	99.7	99.9	96.8	80.0
Section	7	Filtered	94.9	99.4	99.9	97.2	82.8
Section	8	Not filtered	94.5	99.4	100.0	97.0	80.0
Section	8	Filtered	94.5	99.4	100.0	97.0	80.0

The results indicate that the effect of continent filtering is a modest improvement in identifier performance when filtering was applied to the species identifier. For the section identifier, there was no such improvement for CG8. Sections are less likely to be restricted to any given continent, so there are fewer results to filter out, and for sections which are restricted by continent, the identifier did not make any mistakes.

Whilst analysing the results of one identifier it was observed that significant errors could occur for *individual* collections when filtering was applied. Specifically, due to the small number of *H. grandisporum* collections and the random nature of the set selection, only collections from North America (Greenland) happened to be included in the training set, whereas the testing set included a collection from Europe. The identifier with continent filtering applied thus always assigned zero probability to this collection being *H. grandisporum–*despite its characteristic large spores–as, from the perspective of the identifier, that species was “unknown” in Europe.

Table 8 shows the choice of functional optimizer makes a small difference to the accuracy of the identifier especially in the more difficult case of species identification. Overall the AMSGrad variant of the AdamW optimizer achieved the best results across all metrics and identifier. The results presented here are consistent with the general machine learning literature in which AdamW with AMSGrad was presented as in improvement over AdamW which in turn was presented as an improvement over Adam, suggesting that the species identification problem may stand to benefit from any further improvements that are made by the machine learning specialists. For classification to section, the differences were very small.

**Table 8:** Comparison of results with different functional optimizers

			Metrics (/100)				
Class	CG	Optimiser	Top 1	Top 3	Top 5	MRR	F1m
Species	7	AdamW (AMSGrad)	75.6	95.7	98.8	85.8	73.3
Species	7	AdamW (original)	75.9	95.7	98.4	85.7	73.0
Species	7	Adam	75.0	95.5	98.8	85.2	72.8
Species	7	SGD	74.7	93.9	97.0	84.3	70.8
Species	8	AdamW (AMSGrad)	77.7	96.2	99.4	87.1	73.5
Species	8	AdamW (original)	75.0	94.7	98.9	85.2	69.5
Species	8	Adam	76.7	95.8	99.2	86.5	72.2
Species	8	SGD	75.2	93.4	97.7	84.9	70.6
Section	7	AdamW (AMSGrad)	94.9	99.4	99.9	97.2	82.8
Section	7	AdamW (original)	95.4	99.3	99.7	97.4	83.5
Section	7	Adam	94.9	99.3	99.9	97.2	83.0
Section	7	SGD	95.1	99.6	99.9	97.3	83.2
Section	8	AdamW (AMSGrad)	94.5	99.4	100.0	97.0	80.0
Section	8	AdamW (original)	95.1	99.4	99.8	97.3	81.1
Section	8	Adam	94.3	99.4	100.0	97.0	79.8
Section	8	SGD	94.7	99.4	100.0	97.1	81.6

Table 9 shows a mixed picture when considering network shapes and activation functions. For species identification, the one-layer networks (ReLU or Mish alone) performed marginally better than the two-layer variants. This result is initially a little surprising as any one-layer network can be embedded in a two-layer network by setting the second layer to the identity function and so any two-layer network can find at least as good a solution to the optimization problem as a one-layer network. The result here is thus suggestive that the extra free parameters in the two-layer network allow for some overfitting of the training set, and consequently slightly worse results for the testing set. In other words, there is an insufficient quantity of data to solve effectively for all the hidden parameters in a multi-layer network.

**Table 9:** Summary of results for identifiers with different neural network shapes and activation functions

			Metrics (/100)				
Class	CG	Activation	Top 1	Top 3	Top 5	MRR	F1m
Species	7	ReLU	75.0	95.4	98.8	85.2	72.4
Species	7	Mish	75.7	95.1	98.8	85.7	72.9
Species	7	ReLU/ReLU	72.7	94.9	97.9	84.1	72.2
Species	7	ReLU/Mish	75.1	95.8	98.1	85.4	72.9
Species	7	Mish/ReLU	74.4	95.8	98.2	84.8	71.6
Species	7	Mish/Mish	75.3	95.8	98.4	85.5	72.3
Species	8	ReLU	76.7	95.8	99.2	86.5	72.1
Species	8	Mish	76.5	95.3	99.1	86.2	72.7
Species	8	ReLU/ReLU	75.8	94.9	97.9	85.5	69.5
Species	8	ReLU/Mish	75.9	95.3	98.5	85.7	71.2
Species	8	Mish/ReLU	75.9	94.7	97.7	85.6	71.2
Species	8	Mish/Mish	75.2	95.3	98.1	85.4	70.0
Section	7	ReLU	94.9	99.3	99.9	97.2	82.8
Section	7	Mish	95.2	99.3	99.9	97.3	83.5
Section	7	ReLU/ReLU	95.1	99.1	99.7	97.2	81.2
Section	7	ReLU/Mish	94.9	99.4	99.7	97.2	83.1
Section	7	Mish/ReLU	94.9	99.0	100.0	97.1	80.8
Section	7	Mish/Mish	94.6	99.1	99.9	97.0	80.7
Section	8	ReLU	94.5	99.4	100.0	97.0	80.0
Section	8	Mish	94.1	99.6	100.0	96.9	78.3
Section	8	ReLU/ReLU	93.8	99.4	99.8	96.6	79.0
Section	8	ReLU/Mish	94.5	99.6	99.8	97.0	78.7
Section	8	Mish/ReLU	94.1	99.4	99.8	96.8	78.8
Section	8	Mish/Mish	94.5	99.6	99.8	97.0	78.3

## DISCUSSION

The results presented here indicate that the approach of using character-based information as input data to a machine learning algorithm shows significant promise. The character group that yields best results (Character Group 8) only requires 17 pieces of information, leading to a feature vector dimension of 32 from a training set of just over 2,000 collections to produce an identifier that identifies the correct species 76% of the time, and over 99% of the time the correct species is within the top 5 ‘guesses’ produced by the identifier (from a list of 123). Somewhat remarkably, armed with locality data and microscopic data relating to spore size and the size and shape of cheilocystidia, CG8 only requires one piece of macroscopic data, that of the number of full-length lamellae, in order to make its determination.

The problem of identifying the correct (sub)section is inherently easier: there are a total of 21 output classes for the identifier to select from in this case, rather than 123, but the training set is the same size. The size of the training set per output class is thus higher, the identifier effectively has more information and consequently it performs better: the correct section is chosen in 95% of the cases. Over 99% of the time, the identifier puts the correct section in its top 3 “guesses”.

In practical terms, we believe the identifier is relatively user-friendly. Although the identifier does demand a number of microscopic measurements and determinations, these are characters that a competent mycologist should be able to determine. A user interface to the identifier will be available on the website, https://hebeloma.org (Bartlett et al. 2021). Given the very small improvement in accuracy of CG8 to CG7, we have chosen to present CG7 on the website; this absolves the user from the extra task of calculating basidia Q.

The interface will be familiar to users of multi-access keys. Unlike a decision tree, where the user inputs one piece of information at a time, all characters are provided at once. The answer is returned more-or-less immediately (whilst the calibration of an identifier takes a minute or two on a standard computer, once calibrated, the identification function is very quick to compute). Whereas multi-access keys tend to return one result, or at best a list of equiprobable results, the identifier approach allows the possibilities to be ranked in probabilistic order. We show the Top 5 results and the user can then study the species descriptions of the five presented options and build confidence (or otherwise!) in the determination that the machine has made. Across all the identifiers tried, the performance was generally insensitive to the metric used–i.e. if a species identifier performs well according to one metric, it also tended to perform well according to the other metrics, relative to other identifiers, so using “Top 5” in the user interface seems reasonable.

While we think the results are already good enough to provide value to mycologists attempting a *Hebeloma* identification, there are two reasons to hope that the results will continue to get better over time as more collections are added to our dataset. Firstly, this is what tends to happen in general for supervised learning problems–e.g. problems of email spam detection or image recognition benefit from additional data, and we would hope for the same in the *Hebeloma* case. Secondly, and more specifically, we conducted an experiment where we ignored the existence of the 23 species with the fewest available collections and concentrated only on the remaining 100. In this case, the number of “Top 1” guesses rose from 76 to 80%. This is a proxy (albeit not a perfect one) for what we might expect to happen as we gather more collections.

Thus, there are two structural advantages of this type of identifier over a classic multi-access key–the ability to rank and weight suggestions (and so have “Top 3” and “Top 5” metrics) rather than just having the “Top 1” implied by a key and also the ability to adapt automatically over time as extra information is obtained–classical keys tend to have to be refined manually. Nevertheless, it is intriguing to set aside the structural differences and simply compare our identifier’s Top 1 score of 76% with a similar result for a classical key. To do this, we looked at all collections in our database dated after 2016; 2016 was chosen as only after this date were the keys of Beker et al. (2016) generally available. This was a total of 2500 collections. Of these just 339 collections had been identified to species, i.e. 14%, and of these 339, 179 (7% of the total and 53% of those assigned a species) were in accordance with our determinations based on morphological and molecular results as well as habitat and locality. Although this method for estimating the accuracy of a key is imperfect (we do not know exactly how determiners made their determination), it does give some confidence that the approach presented here is valuable.

It is encouraging that best results were achieved with a relatively “out-of-the-box” neural network. Only one hidden layer was applied and the common ReLU activation function and AdamW solver were used. The computation effort was relatively modest: On a typical laptop and for a given choice of Character Group, activation function and optimizer, the software takes approximately two minutes to find the optimal network for each learning rate, and then to choose the learning rate which gives most performant results. The script has a peak memory usage of approximately 300 MB when processing a training set of 2,000 collections.

Data augmentation in the form of interpolating missing data was helpful in most of the cases we tested–the result shown in Table 5 where CG7 results were worse with augmented was actually relatively unusual. The case of CG8, when augmentation improved the identifier, was more typical in our experience. However, our results showed that the extension idea of posthoc filtering was not, overall, beneficial. The idea of adding specific identifiers for specific subsections did not appear to give improvements. The most likely cause of this is the relatively small number of collections available for the second pass dedicated to the *Hebeloma* ‘subsect 1’ section was too small to train the identifier properly. It is, however, our opinion that the idea of section-specific identifiers is worth pursuing as more collections become available to us. Whilst here we applied the idea to only one sub-section with an adequate number of collections, it seems possible that each section could have its own identifier. Setting a loftier goal, it may even be possible to use multiple chained character-based identifiers to classify a collection from a range of possible genera.

Our approach uses characters to guide the machine learning algorithm. It is more common in the literature to use images. Image-based algorithms however usually discretize images to 224 × 224 or 256 × 256 pixels (ImageNet, Deng et al. 2009) and each pixel is effectively an input dimension. Therefore, the overall dimensionality and thus computational cost to provide an identification of an image-based identifier is likely to be significantly higher than the character-based one presented here. Given the broad macroscopic similarity among *Hebeloma* species, this extra computation may not pay dividends, at least for macroscopic images.

A natural question is whether the supervised learning approach followed here could be extended to other genera and families. In principle there appears to be no reason why not, at least in the cases where species boundaries have (also) been determined by morphology. For *Hebeloma*, we have possibly relied more on morphology than colleagues working on other groups of fungi (e.g. Quaedvlieg et al. 2014; Bazzicalupo et al. 2017; Sato et al. 2020).

Traditional binomial, multinomial and synoptic keys are special cases of the mappings from characters to species that a machine learning algorithm can ‘discover’ and so we anticipate that the approach could work for genera other than *Hebeloma*, given sufficient data. We should emphasize however that the learning was *supervised* in two senses: Firstly, the species boundaries were already delineated prior to commencing this work. Secondly, the character groups that the machine operated on were defined in advance, utilizing considerable experience in knowing which characters would be useful to help the machine “find” the species boundaries, and then applying a degree of manual trial-and-error to find the nine character groups presented here. An intriguing topic of future research would be to extend the ideas here to pass all available character information to the machine and allow it to ‘self-discover’ an appropriate group of characters. A simpler approach might be to take an existing key for a given genus or family and simply take all characters used in the key as the character group to the machine.

In experimenting with machine learning tools applied to characters, our experience has been that the tools performed best when provided with objective information: most of the features (GPS coordinates, average spore and cystidia measurements, lamellae count) are unambiguous. Spore features and cheilocystidia main shape are more subjective but appear crucial to species determination in *Hebeloma* and so appear in our best-performing identifiers.

With this subjectivity in mind, it seems wise to ask whether the very good results observed are too good. We find that the identifier can be very sensitive to its inputs, particularly the discretely-valued inputs spore ornamentation and cheilocystidia shape. Table 10 shows a typical example of such sensitivity. Ordinarily in machine learning, this high sensitivity would suggest a degree of overfitting. However, the pattern persists even when cross-validating training sets. Because many of the collections in the database were analysed and assigned characters by us, the phenomenon is perhaps arising because of the very high degree of consistency in assigning discrete characters. There is also an appreciable possibility of unconscious bias on behalf of the assigning expert if the species and characters are assigned at the same time. In practice there will be a degree of subjectivity in assigning these properties that may vary from one mycologist to the next, that our identifier currently has no ability to take into account. I.e., there is a risk that our classifier is currently calibrated to ‘characters as assessed and assigned by us’, and will not work as effectively when classifying the collections assessed by others.

**Table 10:** The outputs of the identifier for the collection with parameters shown in Fig. 2 but now with the spore ornamentation (only) varied. As the ornamentation is identified as O3 instead of O2, the identification swings significantly from *Hebeloma circinans* to *H. pumilum*

	Spore ornamentation		
Species	O2 (%)	O2; O3 (%)	O3 (%)
*H. circinans*	50.6	67.4	21.0
*H. nanum*	12.4	3.1	3.8
*H. lindae*	5.9	< 1	< 1
*H. cylindrosporum*	5.4	< 1	< 1
*H. naviculosporum*	5.0	2.1	4.5
*H. pseudoamarescens*	3.3	< 1	2.20
*H. pumilum*	2.8	12.5	31.5
*H. danicum*	1.1	2.7	12.6
*H. laterinum*	< 1	1.2	9.1

As stated earlier, however, the general principle of a character-based identifier does show significant promise especially for a genus like *Hebeloma* where a macroscopic image-based identifier would struggle to distinguish between species. Therefore, if the identifier is over-sensitive to this ‘assessor’ problem, it should be possible to ameliorate it by training it on more data assessed by a wider range of experts.

Another limitation of the machine learning-based approach is that whilst it answers the ‘what’ question, it does not answer the’why’ question. That is, the algorithm says which species it thinks a collection is likely to be, but it does not say why it is that species as opposed to any other and does not say which characters were particularly significant. This contrasts, for example, with a traditional multi-access key or decision tree where the ‘line of reasoning’ can be completely followed.

This study is an attempt to classify species based solely on applying machine learning to quantified characters. As noted in the introduction, however, it is more common in the literature to attempt classification using images and computer vision, whether this be microscopic images (Zieliński et al. 2020) or macroscopic (Šulc et al. 2020). Picek et al. (2021) showed that starting from image data and then adding what they call metadata, similar to our quantified characters, could realize improvements in recognition. It would make sense therefore for us to act in the other direction–start with characters and see if improvement can be made by adding images. We propose investigating this in future work.

This study also made no attempt to utilize the ITS sequences available for most of the collections on our database. An intriguing possibility would be to combine the ideas of Delgado-Serrano et al. (2016) and those of this paper to create an identifier that uses sequence and morphological data simultaneously. We intend to make the website https://hebeloma.org (Bartlett et al. 2021, in prep.) available that allows the user to test the indentifier against *Hebeloma* data provided by us or by the user. We hope that others will take the identifier as proposed here up and apply it to other groups of organisms.

## CONCLUSIONS

This paper introduces a novel way to attempt identifying collections to species or section. The results presented here indicate that the approach of using character-based information as input data to a machine learning algorithm shows significant promise. This novel approach to species identification could theoretically be used for any genus where sufficient data exists to train the machine and allow it to select the ‘best’ algorithm. As demonstrated, this technique not only achieves good accuracy in identifying the species of a given collection but also has huge advantages over traditional single-access key and multi-access keys. In addition to being able to seek out an optimal mapping between characters and species, it is also able to automatically learn and adapt to changes, such as the introduction of new species or even to changes in species boundaries as more collections of a species become available and the definition widens. Here, *Hebeloma* has been used as a case study, but the genus is not exceptional, other than through the presence of a large amount of data that can be applied to the machine-learning. The technique should be replicable to other genera. A user-friendly interface to the *Hebeloma* identifier will be made available shortly on https://hebeloma.org.

## Supplementary Information

**Additional file 1**: **Appendix Fig. S1.** The probability distribution of each of the continuously-valued characters used in one or more of the identifiers. The probability distribution also acts as a mapping from the character to a feature value between 0 and 1. The characters are **a** latitude, **b** longitude, **c** altitude, **d** number of complete lamellae, **e** average spore length, **f** average spore width, **g** average spore Q, **h** average cheilocystidia length, **i** average cheilocystidia width, **j** average cheilocystidia A/M (width at apex/width at narrowest point in central region), k average cheilocystidia A/B (width at apex/maximum width at lower third), **l** average cheilocystidia B/M (maximum width at lower third/ width at narrowest point in central region), **m** basidia Q, and **n** stipe width.

**Additional file 2**: **Appendix Table S1.** Results of the identifier using characters from Character Group 7 and standard parameters as in Table 3. For each species, the number of collections that were identified correctly in the identifiers top 5 ‘guesses’ are shown. If a species had any incorrect guesses (i.e. any guess that was not Top 1, then the top guess of the identifier is shown in the ‘Confused as’ column. Species shown in bold in the ‘Confused as’ column are from the same (sub)-section. Species not shown in bold are from different (sub)-sections.

## Data Availability

The computer code for creating identifiers and verifying results is available at https://github.com/Pcb21/hebeloma-project/tree/main/hebident. The software package is licenced under GNU GPL vs. 0.3. Collection data will be available on the authors’ website, https://hebeloma.org.

## Footnotes

1 We here refer by “ITS” to the amplicon of the ITS region by standard fungal ITS primers, including the ITS1, the 5.8S rDNA, the ITS2 and in many cases also short stretches of the 5’-end of the SSU and of the 3’-end of the LSU of the nuclear ribosomal genes.

2 In this paper ML will exclusively be used to abbreviate Machine Learning, not Maximum Likelihood.
