# Supplementary material for: Species determination using AI machine-learning algorithms: Hebeloma as a case study
Source: IMA Fungus. 2022 Jun 30;13:13. doi: 10.1186/s43008-022-00099-x (PMC9245212; doi:10.1186/s43008-022-00099-x)
Supplement: Supplementary file 1 — Additional file 1: Appendix Fig. S1. The probability distribution of each of the continuously-valued characters used in one or more of the identifiers. The probability distribution also acts as a mapping from the character to a feature value between 0 and 1. The characters are a latitude, b longitude, c altitude, d number of complete lamellae, e average spore length, f average spore width, g average spore Q, h average cheilocystidia length, i average cheilocystidia width, j average cheilocystidia A/M (width at apex/width at narrowest point in central region), k average cheilocystidia A/B (width at apex/maximum width at lower third), l average cheilocystidia B/M (maximum width at lower third/ width at narrowest point in central region), m basidia Q, and n stipe width. [file 43008_2022_99_MOESM1_ESM.pdf]

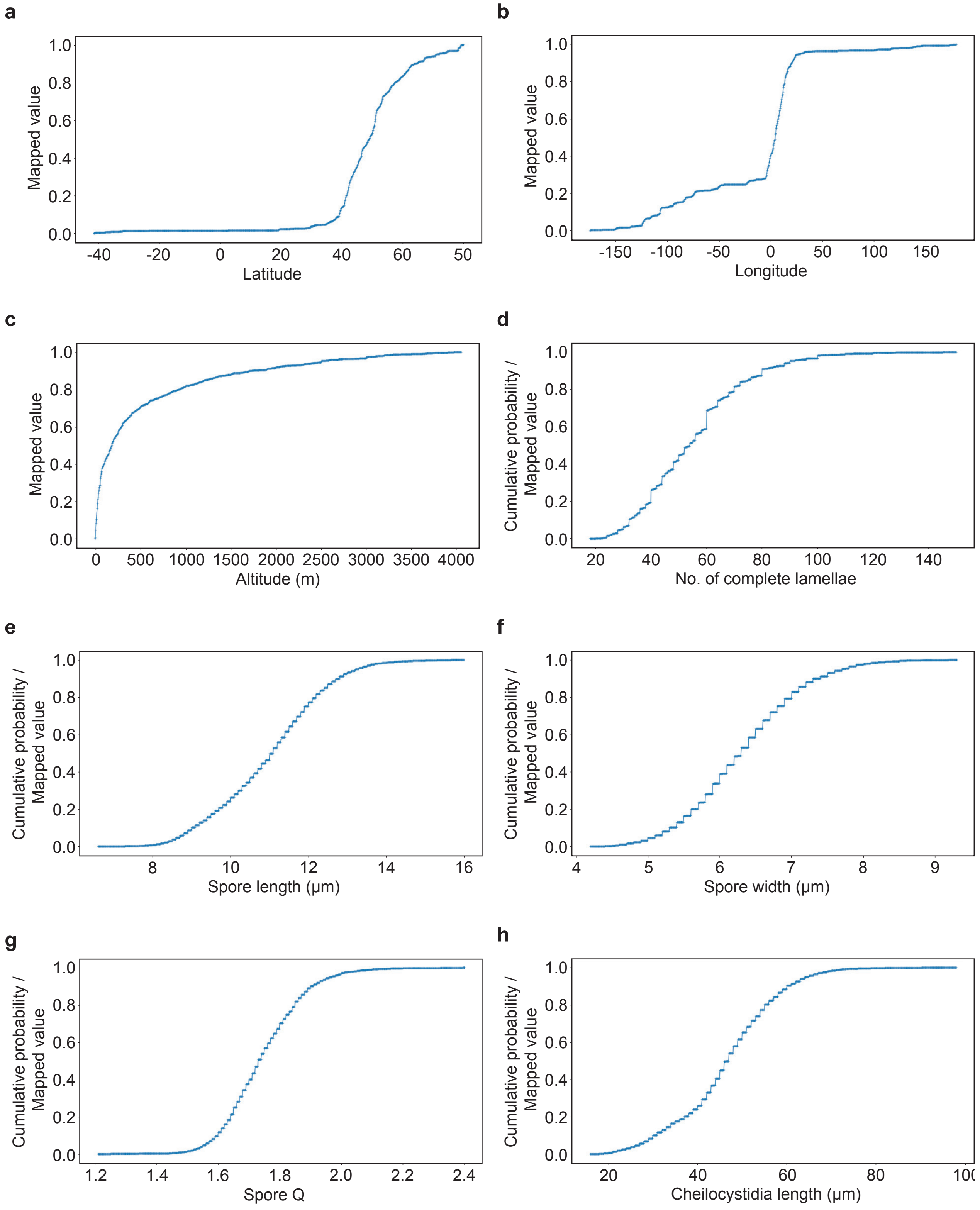

Appendix Fig. S1. The probability distribution of each of the continuously-valued characters used in one or more of the identifiers. The probability distribution also acts as a mapping from the character to a feature value between 0 and 1. The characters are **a** latitude, **b** longitude, **c** altitude, **d** number of complete lamellae, **e** average spore length, **f** average spore width, **g** average spore Q, **h** average cheilocystidia length, **i** average cheilocystidia width, **j** average cheilocystidia A/M (width at apex/width at narrowest point in central region), **k** average cheilocystidia A/B (width at apex/maximum width at lower third), **l** average cheilocystidia B/M (maximum width at lower third/ width at narrowest point in central region), **m** basidia Q, and **n** stipe width.

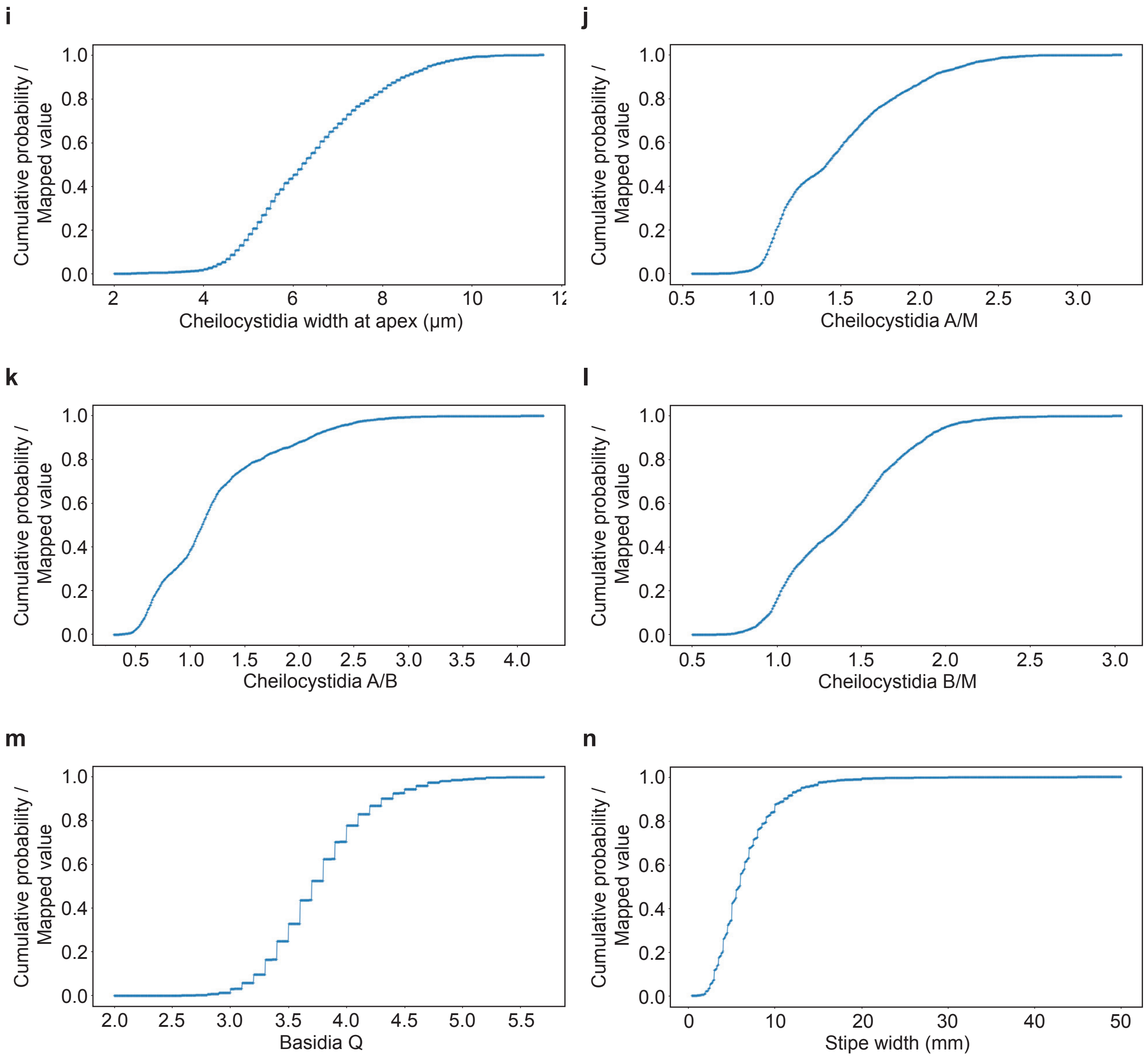

Appendix Fig. S1 – continued.
