# Supplementary material for: Species determination using AI machine-learning algorithms: Hebeloma as a case study
Source: IMA Fungus. 2022 Jun 30;13:13. doi: 10.1186/s43008-022-00099-x (PMC9245212; doi:10.1186/s43008-022-00099-x)
Supplement: Supplementary file 2 — Additional file 2: Appendix Table S1. Results of the identifier using characters from Character Group 7 and standard parameters as in Table 3. For each species, the number of collections that were identified correctly in the identifiers top 5 ‘guesses’ are shown. If a species had any incorrect guesses (i.e. any guess that was not Top 1, then the top guess of the identifier is shown in the ‘Confused as’ column. Species shown in bold in the ‘Confused as’ column are from the same (sub)-section. Species not shown in bold are from different (sub)-sections. [file 43008_2022_99_MOESM2_ESM.pdf]

**Appendix Table S1.** Results of the identifier using characters from Character Group 7 and standard parameters as in Table 3. For each species, the number of collections that were identified correctly in the identifiers top 5 'guesses' are shown. If a species had any incorrect guesses (i.e. any guess that was not Top 1, then the top guess of the identifier is shown in the 'Confused as' column. Species shown in bold in the 'Confused as' column are from the same (sub)-section. Species not shown in bold are from different (sub)-sections.

| ***Adherentia*** | **Total** | **1st** | **2nd** | **3rd** | **4th** | **5th** | **MRR score** | **Confused as** |
|---|---|---|---|---|---|---|---|---|
| *H. adherens* | 1 | 0 | 0 | 0 | 1 | 0 | 25 | *H. radicosum* (1) |
| **Total** | **1** | **0 (0%)** | **0 (0%)** | **0 (0%)** | **1 (100%)** | **0 (0%)** | **25** | |

| **'Australe'** | **Total** | **1st** | **2nd** | **3rd** | **4th** | **5th** | **MRR score** | **Confused as** |
|---|---|---|---|---|---|---|---|---|
| *H. australe* | 6 | 6 | 0 | 0 | 0 | 0 | 100 | |
| **Total** | **6** | **6 (100%)** | **0 (0%)** | **0 (0%)** | **0 (0%)** | **0 (0%)** | **100** | |

| ***Denudata-Clepsydroida*** | **Total** | **1st** | **2nd** | **3rd** | **4th** | **5th** | **MRR score** | **Confused as** |
|---|---|---|---|---|---|---|---|---|
| *H. album* | 11 | 4 | 4 | 2 | 1 | 0 | 62 | *H. lutense* (1), ***H. cavipes*** (1), *H. hiemale* (1), ***H. ingratum*** (1), ***H. limbatum*** (1), ***H. sordidulum*** (1), ***H. vaccinum*** (1) |
| *H. ammophilum* | 2 | 1 | 0 | 1 | 0 | 0 | 66 | *H. velutipes* (1) |
| *H. asperosporum* | 1 | 1 | 0 | 0 | 0 | 0 | 100 | |
| *H. cavipes* | 17 | 13 | 2 | 2 | 0 | 0 | 86 | ***H. limbatum*** (1), ***H. album*** (1), ***H. sordidulum*** (1), ***H. ingratum*** (1) |
| *H. cinnamomeum* | 3 | 1 | 2 | 0 | 0 | 0 | 66 | ***H. cavipes*** (1), *H. hiemale* (1) |
| *H. ingratum* | 4 | 2 | 1 | 0 | 1 | 0 | 68 | ***H. pseudofragilipes*** (1), ***H. album*** (1) |
| *H. limbatum* | 3 | 1 | 1 | 0 | 0 | 1 | 56 | *H. celatum* (1), ***H. pseudofragilipes*** (1) |
| *H. matritense* | 1 | 0 | 0 | 0 | 1 | 0 | 25 | ***H. ammophilum*** (1) |
| *H. pseudofragilipes* | 12 | 6 | 2 | 1 | 0 | 1 | 64 | ***H. cavipes*** (3), ***H. album*** (1), ***H. ingratum*** (1), *H. hiemale* (1) |
| *H. sordidulum* | 12 | 12 | 0 | 0 | 0 | 0 | 100 | |
| *H. vaccinum* | 11 | 10 | 1 | 0 | 0 | 0 | 95 | *H. pusillum* (1) |
| **Total** | **77** | **51 (66%)** | **13 (17%)** | **6 (8%)** | **3 (4%)** | **2 (3%)** | **78** | |

| ***Denudata-Crustuliniformia*** | **Total** | **1st** | **2nd** | **3rd** | **4th** | **5th** | **MRR score** | **Confused as** |
|---|---|---|---|---|---|---|---|---|
| *H. aanenii* | 12 | 7 | 5 | 0 | 0 | 0 | 79 | ***H. geminatum*** (5) |
| *H. alpinum* | 24 | 19 | 3 | 1 | 0 | 0 | 87 | ***H. eburneum*** (2), *H. hiemale* (2), ***H. crustuliniforme*** (1) |
| *H. arcticum* | 3 | 3 | 0 | 0 | 0 | 0 | 100 | |
| *H. aurantioumbrinum* | 5 | 4 | 1 | 0 | 0 | 0 | 90 | *H. hiemale* (1) |
| *H. bellotianum* | 0 | 0 | 0 | 0 | 0 | 0 | - | |
| *H. crustuliniforme* | 8 | 5 | 2 | 0 | 1 | 0 | 78 | ***H. eburneum*** (2), ***H. aanenii*** (1) |
| *H. eburneum* | 27 | 21 | 4 | 2 | 0 | 0 | 87 | ***H. alpinum*** (3), *H. album* (1), ***H. lutense*** (1), ***H. luteicystidiatum*** (1) |
| *H. geminatum* | 9 | 8 | 1 | 0 | 0 | 0 | 94 | ***H. aanenii*** (1) |
| *H. helodes* | 10 | 10 | 0 | 0 | 0 | 0 | 100 | |
| *H. louiseae* | 2 | 1 | 0 | 1 | 0 | 0 | 66 | ***H. aurantioumbrinum*** (1) |
| *H. luteicystidiatum* | 1 | 0 | 0 | 1 | 0 | 0 | 33 | ***H. louiseae*** (1) |
| *H. lutense* | 5 | 5 | 0 | 0 | 0 | 0 | 100 | |
| *H. magnicystidiatum* | 0 | 0 | 0 | 0 | 0 | 0 | - | |
| *H. minus* | 6 | 5 | 1 | 0 | 0 | 0 | 91 | ***H. pusillum*** (1) |
| *H. pallidolabiatum* | 2 | 2 | 0 | 0 | 0 | 0 | 100 | |
| *H. perexiguum* | 0 | 0 | 0 | 0 | 0 | 0 | - | |
| *H. pusillum* | 6 | 5 | 0 | 0 | 0 | 0 | 85 | *H. paludicola* (1) |
| *H. salicicola* | 8 | 8 | 0 | 0 | 0 | 0 | 100 | |
| **Total** | **128** | **103 (80%)** | **17 (13%)** | **5 (4%)** | **1 (1%)** | **0 (0%)** | **88** | |

| ***Denudata-Echinospora*** | **Total** | **1st** | **2nd** | **3rd** | **4th** | **5th** | **MRR score** | **Confused as** |
|---|---|---|---|---|---|---|---|---|
| *H. echinosporum* | 0 | 0 | 0 | 0 | 0 | 0 | - | |
| *H. populinum* | 1 | 1 | 0 | 0 | 0 | 0 | 100 | |
| *H. rostratum* | 4 | 3 | 1 | 0 | 0 | 0 | 87 | ***H. populinum*** (1) |
| **Total** | **5** | **4 (80%)** | **1 (20%)** | **0 (0%)** | **0 (0%)** | **0 (0%)** | **89** | |

| ***Denudata-Hiemalia*** | **Total** | **1st** | **2nd** | **3rd** | **4th** | **5th** | **MRR score** | **Confused as** |
|---|---|---|---|---|---|---|---|---|
| *H. hiemale* | 19 | 18 | 0 | 1 | 0 | 0 | 96 | *H. crustuliniforme* (1) |
| **Total** | **19** | **18 (95%)** | **0 (0%)** | **1 (5%)** | **0 (0%)** | **0 (0%)** | **96** | |

| ***Duracinus*** | **Total** | **1st** | **2nd** | **3rd** | **4th** | **5th** | **MRR score** | **Confused as** |
|---|---|---|---|---|---|---|---|---|
| *H. duracinoides* | 2 | 2 | 0 | 0 | 0 | 0 | 100 | |
| **Total** | **2** | **2 (100%)** | **0 (0%)** | **0 (0%)** | **0 (0%)** | **0 (0%)** | **100** | |

| ***Hebeloma*-'subsect1'** | **Total** | **1st** | **2nd** | **3rd** | **4th** | **5th** | **MRR score** | **Confused as** |
|---|---|---|---|---|---|---|---|---|
| *H. fuscatum* | 5 | 3 | 1 | 1 | 0 | 0 | 76 | ***H. nigellum*** (1), ***H. paludicola*** (1) |
| *H. grandisporum* | 1 | 0 | 0 | 1 | 0 | 0 | 33 | ***H. spetsbergense*** (1) |
| *H. monticola* | 6 | 5 | 1 | 0 | 0 | 0 | 91 | ***H. palustre*** (1) |
| *H. nigellum* | 7 | 3 | 3 | 1 | 0 | 0 | 69 | ***H. fuscatum*** (3), ***H. paludicola*** (1) |
| *H. paludicola* | 8 | 5 | 0 | 1 | 1 | 0 | 71 | ***H. fuscatum*** (3) |
| *H. palustre* | 24 | 23 | 1 | 0 | 0 | 0 | 97 | ***H. monticola*** (1) |
| *H. sordescens* | 5 | 2 | 1 | 1 | 0 | 0 | 60 | ***H. palustre*** (2), ***H. monticola*** (1) |
| *H. spetsbergense* | 14 | 8 | 3 | 1 | 0 | 0 | 72 | ***H. fuscatum*** (3), ***H. nigellum*** (2), ***H. paludicola*** (1) |
| **Total** | **70** | **49 (70%)** | **10 (14%)** | **6 (9%)** | **1 (1%)** | **0 (0%)** | **80** | |

| ***Hebeloma*-'subsect2'** | **Total** | **1st** | **2nd** | **3rd** | **4th** | **5th** | **MRR score** | **Confused as** |
|---|---|---|---|---|---|---|---|---|
| *H. alpinicola* | 15 | 8 | 2 | 1 | 1 | 0 | 66 | ***H. excedens*** (4), ***H. mesophaeum*** (3) |
| *H. ambustiterranum* | 6 | 6 | 0 | 0 | 0 | 0 | 100 | |
| *H. cistophilum* | 6 | 5 | 1 | 0 | 0 | 0 | 91 | ***H. subtortum*** (1) |
| *H. colvinii* | 3 | 3 | 0 | 0 | 0 | 0 | 100 | |
| *H. excedens* | 14 | 4 | 6 | 3 | 1 | 0 | 58 | ***H. alpinicola*** (5), ***H. mesophaeum*** (3), ***H. velatum*** (2) |
| *H. harperi* | 2 | 2 | 0 | 0 | 0 | 0 | 100 | |

| ***Hebeloma*-'subsect2'** continued | **Total** | **1st** | **2nd** | **3rd** | **4th** | **5th** | **MRR score** | **Confused as** |
|---|---|---|---|---|---|---|---|---|
| *H. marginatulum* | 6 | 4 | 1 | 0 | 0 | 1 | 78 | ***H. alpinicola*** (1), ***H. mesophaeum*** (1) |
| *H. mesophaeum* | 16 | 12 | 3 | 1 | 0 | 0 | 86 | ***H. alpinicola*** (2), ***H. excedens*** (2) |
| *H. pascuense* | 1 | 0 | 0 | 0 | 1 | 0 | 25 | ***H. excedens*** (1) |
| *H. psammophilum* | 2 | 1 | 1 | 0 | 0 | 0 | 75 | ***H. cistophilum*** (1) |
| *H. pubescens* | 2 | 2 | 0 | 0 | 0 | 0 | 100 | |
| *H. subtortum* | 7 | 5 | 2 | 0 | 0 | 0 | 85 | ***H. mesophaeum*** (2) |
| *H. velatum* | 14 | 13 | 1 | 0 | 0 | 0 | 96 | ***H. marginatulum*** (1) |
| **Total** | **94** | **65 (69%)** | **17 (18%)** | **5 (5%)** | **3 (3%)** | **1 (1%)** | **80** | |

| ***'Islandica'*** | **Total** | **1st** | **2nd** | **3rd** | **4th** | **5th** | **MRR score** | **Confused as** |
|---|---|---|---|---|---|---|---|---|
| *H. islandicum* | 1 | 0 | 0 | 0 | 0 | 0 | 9 | *H. pallidolabiatum* (1) |
| **Total** | **1** | **0 (0%)** | **0 (0%)** | **0 (0%)** | **0 (0%)** | **0 (0%)** | **9** | |

| ***'Mediorufa'*** | **Total** | **1st** | **2nd** | **3rd** | **4th** | **5th** | **MRR score** | **Confused as** |
|---|---|---|---|---|---|---|---|---|
| *H. lacteocoffeatum* | 0 | 0 | 0 | 0 | 0 | 0 | - | |
| *H. mediorufum* | 0 | 0 | 0 | 0 | 0 | 0 | - | |
| *H. nothofagetorum* | 0 | 0 | 0 | 0 | 0 | 0 | - | |
| **Total** | **0** | **0** | **0 (0%)** | **0 (0%)** | **0 (0%)** | **0 (0%)** | **-** | |

| ***Myxocybe*** | **Total** | **1st** | **2nd** | **3rd** | **4th** | **5th** | **MRR score** | **Confused as** |
|---|---|---|---|---|---|---|---|---|
| *H. radicosum* | 4 | 4 | 0 | 0 | 0 | 0 | 100 | |
| *H. sagarae* | 1 | 1 | 0 | 0 | 0 | 0 | 100 | |
| **Total** | **5** | **5 (100%)** | **0 (0%)** | **0 (0%)** | **0 (0%)** | **0 (0%)** | **100** | |

| ***Naviculospora*** | **Total** | **1st** | **2nd** | **3rd** | **4th** | **5th** | **MRR score** | **Confused as** |
|---|---|---|---|---|---|---|---|---|
| *H. avellaneum* | 3 | 3 | 0 | 0 | 0 | 0 | 100 | |
| *H. catalaunicum* | 2 | 1 | 1 | 0 | 0 | 0 | 75 | *H. velutipes* (1) |
| *H. nanum* | 5 | 2 | 2 | 0 | 0 | 1 | 64 | *H. cylindrosporum* (1), *H. circinans* (1), ***H. catalaunicum*** (1) |
| *H. naviculosporum* | 4 | 3 | 0 | 0 | 1 | 0 | 81 | *H. cavipes* (1) |
| *H. subaustrale* | 2 | 2 | 0 | 0 | 0 | 0 | 100 | |
| *H. subfastibile* | 1 | 1 | 0 | 0 | 0 | 0 | 100 | |
| **Total** | **17** | **12 (71%)** | **3 (18%)** | **0 (0%)** | **1 (6%)** | **1 (6%)** | **82** | |

| ***Porphyrospora*** | **Total** | **1st** | **2nd** | **3rd** | **4th** | **5th** | **MRR score** | **Confused as** |
|---|---|---|---|---|---|---|---|---|
| *H. aminophilum* | 4 | 4 | 0 | 0 | 0 | 0 | 100 | |
| *H. angustilamellatum* | 2 | 1 | 0 | 0 | 1 | 0 | 62 | *H. danicum* (1) |
| *H. flavidifolium* | 1 | 1 | 0 | 0 | 0 | 0 | 100 | |
| *H. ifeleletorum* | 0 | 0 | 0 | 0 | 0 | 0 | - | |
| *H. indicum* | 0 | 0 | 0 | 0 | 0 | 0 | - | |
| *H. lactariolens* | 1 | 0 | 0 | 1 | 0 | 0 | 33 | ***H. youngii*** (1) |
| *H. parvisporum* | 1 | 1 | 0 | 0 | 0 | 0 | 100 | |
| *H. porphyrosporum* | 2 | 2 | 0 | 0 | 0 | 0 | 100 | |
| *H. radicans* | 0 | 0 | 0 | 0 | 0 | 0 | - | |
| *H. sarcophylum* | 0 | 0 | 0 | 0 | 0 | 0 | - | |
| *H. victoriense* | 1 | 1 | 0 | 0 | 0 | 0 | 100 | |
| *H. vinosophyllum* | 1 | 1 | 0 | 0 | 0 | 0 | 100 | |
| *H. youngii* | 0 | 0 | 0 | 0 | 0 | 0 | - | |
| **Total** | **13** | **11 (85%)** | **0 (0%)** | **1 (8%)** | **1 (8%)** | **0 (0%)** | **89** | |

| ***Pseudoamarescens*** | **Total** | **1st** | **2nd** | **3rd** | **4th** | **5th** | **MRR score** | **Confused as** |
|---|---|---|---|---|---|---|---|---|
| *H. pseudoamarescens* | 4 | 4 | 0 | 0 | 0 | 0 | 100 | |
| **Total** | **4** | **4 (100%)** | **0 (0%)** | **0 (0%)** | **0 (0%)** | **0 (0%)** | **100** | |

| ***Sacchariolentia*** | **Total** | **1st** | **2nd** | **3rd** | **4th** | **5th** | **MRR score** | **Confused as** |
|---|---|---|---|---|---|---|---|---|
| *H. fusisporum* | 3 | 2 | 0 | 0 | 1 | 0 | 75 | ***H. ischnostylum*** (1) |
| *H. ischnostylum* | 5 | 4 | 0 | 1 | 0 | 0 | 86 | ***H. fusisporum*** (1) |
| *H. nauseosum* | 5 | 4 | 0 | 1 | 0 | 0 | 86 | *H. pusillum* (1) |
| *H. odoratissimum* | 4 | 0 | 0 | 3 | 0 | 0 | 28 | ***H. nauseosum*** (2),<br>*H. duracinoides* (2) |
| *H. sacchariolens* | 5 | 4 | 1 | 0 | 0 | 0 | 90 | *H. leucosarx* (1) |
| **Total** | **22** | **14 (64%)** | **1 (5%)** | **5 (23%)** | **1 (5%)** | **0 (0%)** | **74** | |

| ***Scabrispora*** | **Total** | **1st** | **2nd** | **3rd** | **4th** | **5th** | **MRR score** | **Confused as** |
|---|---|---|---|---|---|---|---|---|
| *H. anthracophilum* | 5 | 4 | 0 | 0 | 0 | 1 | 84 | *H. ischnostylum* (1) |
| *H. birrus* | 10 | 10 | 0 | 0 | 0 | 0 | 100 | |
| *H. circinans* | 5 | 5 | 0 | 0 | 0 | 0 | 100 | |
| *H. cylindrosporum* | 6 | 5 | 0 | 1 | 0 | 0 | 88 | *H. pseudoamarescens* (1) |
| *H. danicum* | 5 | 5 | 0 | 0 | 0 | 0 | 100 | |
| *H. laterinum* | 12 | 12 | 0 | 0 | 0 | 0 | 100 | |
| *H. lindae* | 3 | 2 | 1 | 0 | 0 | 0 | 83 | *H. quercetorum* (1) |
| *H. luchuense* | 1 | 1 | 0 | 0 | 0 | 0 | 100 | |
| *H. melleum* | 1 | 0 | 1 | 0 | 0 | 0 | 50 | ***H. pumilum*** (1) |
| *H. pumilum* | 6 | 4 | 2 | 0 | 0 | 0 | 83 | ***H. birrus*** (1), ***H. melleum*** (1) |
| *H. radicosoides* | 2 | 2 | 0 | 0 | 0 | 0 | 100 | |
| *H. viscidissimum* | 5 | 4 | 0 | 1 | 0 | 0 | 86 | *H. velutipes* (1) |
| **Total** | **61** | **54 (89%)** | **4 (7%)** | **2 (3%)** | **0 (0%)** | **1 (2%)** | **93** | |

| ***Sinapizantia*** | **Total** | **1st** | **2nd** | **3rd** | **4th** | **5th** | **MRR score** | **Confused as** |
|---|---|---|---|---|---|---|---|---|
| *H. bulbiferum* | 5 | 4 | 1 | 0 | 0 | 0 | 90 | *H. celatum* (1) |
| *H. sinapizans* | 12 | 12 | 0 | 0 | 0 | 0 | 100 | |
| **Total** | **17** | **16 (94%)** | **1 (6%)** | **0 (0%)** | **0 (0%)** | **0 (0%)** | **97** | |

| ***Syrjense*** | **Total** | **1st** | **2nd** | **3rd** | **4th** | **5th** | **MRR score** | **Confused as** |
|---|---|---|---|---|---|---|---|---|
| *H. syrjense* | 6 | 6 | 0 | 0 | 0 | 0 | 100 | |
| **Total** | **6** | **6 (100%)** | **0 (0%)** | **0 (0%)** | **0 (0%)** | **0 (0%)** | **100** | |

| ***Theobromina*** | **Total** | **1st** | **2nd** | **3rd** | **4th** | **5th** | **MRR score** | **Confused as** |
|---|---|---|---|---|---|---|---|---|
| *H. alboerumpens* | 1 | 0 | 1 | 0 | 0 | 0 | 50 | ***H. theobrominum*** (1) |
| *H. cohaerens* | 1 | 1 | 0 | 0 | 0 | 0 | 100 | |
| *H. erumpens* | 2 | 1 | 1 | 0 | 0 | 0 | 75 | ***H. griseopruinatum*** (1) |
| *H. griseopruinatum* | 4 | 3 | 1 | 0 | 0 | 0 | 87 | ***H. erumpens*** (1) |
| *H. parvicystidiatum* | 4 | 4 | 0 | 0 | 0 | 0 | 100 | |
| *H. plesiocistum* | 2 | 2 | 0 | 0 | 0 | 0 | 100 | |
| *H. theobrominum* | 9 | 7 | 0 | 1 | 0 | 0 | 81 | *H. nanum* (2) |
| *H. vesterholtii* | 7 | 4 | 2 | 0 | 0 | 1 | 74 | ***H. theobrominum*** (2), *H. nanum* (1) |
| **Total** | **30** | **22 (73%)** | **5 (17%)** | **1 (3%)** | **0 (0%)** | **1 (3%)** | **83** | |

| ***Velutipes*** | **Total** | **1st** | **2nd** | **3rd** | **4th** | **5th** | **MRR score** | **Confused as** |
|---|---|---|---|---|---|---|---|---|
| *H. aestivale* | 11 | 10 | 1 | 0 | 0 | 0 | 95 | *H. ischnostylum* (1) |
| *H. albidulum* | 7 | 7 | 0 | 0 | 0 | 0 | 100 | |
| *H. celatum* | 6 | 3 | 0 | 2 | 1 | 0 | 65 | ***H. quercetorum*** (2),<br>***H. velutipes*** (1) |
| *H. citrisporum* | 1 | 0 | 1 | 0 | 0 | 0 | 50 | ***H. celatum*** (1) |
| *H. erebium* | 5 | 4 | 1 | 0 | 0 | 0 | 90 | ***H. celatum*** (1) |
| *H. incarnatulum* | 7 | 5 | 2 | 0 | 0 | 0 | 85 | ***H. velutipes*** (2) |
| *H. leucosarx* | 12 | 4 | 6 | 1 | 1 | 0 | 63 | ***H. velutipes*** (8) |
| *H. neurophyllum* | 6 | 6 | 0 | 0 | 0 | 0 | 100 | |
| *H. quercetorum* | 4 | 4 | 0 | 0 | 0 | 0 | 100 | |
| *H. subconcolor* | 3 | 3 | 0 | 0 | 0 | 0 | 100 | |
| *H. velutipes* | 32 | 26 | 6 | 0 | 0 | 0 | 90 | ***H. leucosarx*** (4), *H. alpinum* (1),<br>***H. celatum*** (1) |
| **Total** | **94** | **72 (77%)** | **17 (18%)** | **3 (3%)** | **2 (2%)** | **0 (0%)** | **86** | |
